# Research in Hard-to-Reach Populations: Challenges and Strategies for Conducting Sexual Violence Studies in Applicants for International Protection Beyond the European General Data Protection Regulation

**DOI:** 10.34172/ijhpm.2021.122

**Published:** 2021-08-31

**Authors:** Lotte De Schrijver, Adina Cismaru Inescu, Bastien Hahaut, Christophe Vandeviver, Laurent Nisen, Ines Keygnaert

**Affiliations:** ^1^International Centre for Reproductive Health, Department of Public Health and Primary Care, Ghent University, Ghent, Belgium.; ^2^CARE-ESPRIst, Études et évaluations, University of Liège, Liège, Belgium.; ^3^Institute for International Research on Criminal Policy, Department of Criminology, Criminal Law and Social Law, Ghent University, Ghent, Belgium.; ^4^Research Foundation—Flanders (FWO), Brussels, Belgium.

**Keywords:** Asylum Seekers, Migrant Health, Belgium, Sexual Violence, Privacy Protection, Public Health

## Abstract

**Background:** Conducting research in hard-to-reach populations such as applicants for international protection (AIPs) brings along a number of research challenges. This is especially true for sexual violence (SV) research.

**Methods:** We developed a study design with the intent to reach AIPs in a randomized and anonymous manner including potential illiterate respondents as well, while avoiding as much bias as possible. However, this method was developed just before the entry into force of the new European General Data Protection Regulation (GDPR), upon which important new research challenges emerged.

**Results:** This paper describes the original study design developed to estimate SV prevalence in AIPs in Belgium. We discuss the impact of the GDPR on the recruitment strategy applied to conduct a survey on SV in a randomly selected sample of AIPs, the adapted approach to conduct the study beyond GDPR and lessons learned for future research on sensitive topics in hard-to-reach populations such as AIPs.

**Conclusion:** To achieve reliable prevalence numbers and provide high-quality data on SV in AIPs while respecting the GDPR regulations, studies will require an approach that has become significantly more time consuming and resource-intensive to implement.

## Background

**Key Messages**

** Implications for policy makers**

- Designing General Data Protection Regulation (GDPR) compliant and representative prevalence study designs to measure a sensitive issue such as sexual violence (SV) in hard-to-reach populations requires careful methodological and ethical considerations and a more complex study design. Hence, the current regulations put in place to protect individuals has made it more difficult to reach vulnerable and hard-to-reach populations.
- Compared to previous studies, sufficient project budget should be foreseen to cover the required intensification of the project coordination.
- Collaboration between the research team and stakeholders is crucial and should be facilitated from very early on in the study development stages.
- Larger samples should be drawn to compensate drop-out which is inevitable at every additional step introduced because of GDPR within the recruitment process.
- It is recommended to hire staff specifically assigned to the recruitment process of the study to avoid overburdening the staff in asylum reception centres. Because of GDPR, supplementary steps had to be introduced which involved the staff in the reception centres in every step from sampling to making appointments for interviews. As a result, their involvement was significantly increased.

** Implications for the public**

Sexual violence (SV) is a major public health issue associated with negative consequences in terms of socio-economic wellbeing and physical, mental, sexual and reproductive health. Applicants for international protection (AIPs) appear to be a vulnerable group for SV victimisation. However, robust studies on the prevalence of SV in this population in Belgium are currently lacking. Studying the magnitude and impact of SV in AIPs is crucial in order to develop adequate prevention strategies and care paths focusing on AIPs. Prevention and care are key to increase the wellbeing and integration potential of those affected by SV.

Sexual violence (SV) is a major public health issue with several negative consequences impacting not only individuals, but also larger communities and societies in general. Literature shows that specific hard-to-reach populations such as applicants for international protection (AIPs) are at higher risk of SV exposure than the general population.^1^ The vulnerability for SV in AIPs may be far greater than in the general population because of the specific risk factors that emerge from their vulnerable situation and specific help-seeking barriers associated with their legal status.^2^ Though the risk factors are similar in nature as those found for the general population (ie, age, gender, sexual orientation, socio-economic and health status etc), the impact of sexual victimisation may be greater, and a self-perpetuating circle of increased vulnerability for SV exposure may emerge.^2^ The high number of AIPs in Europe requires the development of prevention strategies and care paths focussing on AIPs exposed to SV.^1^ To this end, a research project started in May 2017 with the aim of improving our UNnderstanding of the MEchanisms, NAture, MAgnitude and Impact of SV in Belgium (UN-MENAMAIS).^3^

 Soon after the project started, on May 25 in 2018, the new General Data Protection Regulation (GDPR) (2016/679) came into force in the European Union (EU).^4^ The GDPR is a regulation and not a directive, which means that although it is immediately binding and applicable, it does provide flexibility to individual member states of the EU to adjust certain aspects of the regulation. Although the GDPR was already adopted on April 14, 2016, how this new regulation should have been applied in the Belgian research context was not yet clear in May 2019. As a result, the original project plan accepted by the donor in 2017 had to be significantly adapted in line with this new legal framework requirements.

 In this paper we will describe the original study design developed to estimate the prevalence of SV in AIPs in Belgium, the impact of the GDPR on the recruitment strategy applied to administer a survey on SV in AIPs, the challenges faced in conducting this study, the strategies applied to conduct the study beyond GDPR and lessons learned for future research on sensitive topics in hard-to-reach populations such as AIPs.

## Methods

###  The Initial Research Strategy

####  Method for Data Collection

 The overall goal of the study was to conduct a quantitative self-report survey probing into SV victimisation and perpetration among a randomly selected sample of AIPs aged 16 to 100 in Belgium, regardless of gender or sexual orientation. Due to ethical and practical considerations related to the legal age of consent in Belgium, only participants having reached the legal age of consent to sex (16+) in Belgium could be considered.

 AIPs are considered a hard-to-reach population because of their high mobility, accessibility through asylum reception centres, frequent restricted internet access and need for assistance when completing an online questionnaire due to language barriers, possible low levels of literacy or limited experience with online tools.^1,5-9^ To increase the response rate among this population and to allow persons with lower literacy levels to participate, we opted for face-to-face interviews to be conducted by trained interviewers. We chose to work with an offline version of the questionnaire – using tablets or laptops – which enabled us to conduct structured interviews on locations with limited internet connection.

 Interviewers speaking at least one of the languages participants were fluent in (cf. infra), were recruited and trained by the research team prior to the start of the interviews. They followed a three-day training for interviewers on talking about violence and sexuality in an intercultural context, on interview techniques, communication skills and the practical aspects related to the use of the questionnaire and the recruitment procedure.

####  Questionnaire Development

 A questionnaire addressing SV victimization and perpetration, including validated scales on SV was designed. To guarantee that respondents with comparable SV experiences would provide similar answers to the questions, behaviourally specific questions were used in the survey.^10-13^ This type of questions avoids bias linked to subjective interpretations of what kinds of behaviours are considered part of SV. Leaving no room for interpretation is crucial to obtain comparable prevalence estimates.^1,10-13^

 The questionnaire was designed to be suitable for structured tablet/laptop assisted personal interviews with AIPs and tested on face validity between August and September 2018. Face validity refers to the extent to which a test is subjectively viewed by test participants as covering the concept it purports to measure. The face validity evaluation process followed two steps:

1. To evaluate the acceptability of the questionnaire in the AIPs population, experts and various members of the target population (ie, from five different age groups between 16 and 100 years old), genders (ie, cisgender female, cisgender male, trans or not-cisgender), sexual orientations (ie, heterosexual and not-heterosexual), migration status (AIPs, refugee, first-generation migrant, second-generation migrant) were asked to fill in the questionnaire and to evaluate the acceptability and phrasing of the questions using a standardized evaluation form. Per target population, 5 to 10 persons were asked to evaluate the questionnaire.
2. Based on the feedback from the experts and members of the target population, the standard questionnaire was adapted in order for the questionnaire to be used efficiently in structured tablet/laptop assisted personal interviews with AIPs. Where needed, instructions for interviewers were added to make the personal interview smoother.

####  Translation and Back-Translation of the Questionnaire

 The questionnaire was (back-)translated into English, French, Dutch, Arabic, Dari, Farsi and Pashtu. The first three languages were chosen as the three most commonly spoken languages in Belgium. We chose to include English instead of German – the third official Belgian language – given that the first is spoken more frequently (often as a second language) in both the Belgian and the AIPs population than the latter. Arabic, Dari, Farsi and Pashtu were specifically chosen to reach a large sample of respondents in a language AIPs are most likely fluent in. Fedasil – the federal agency for the reception of AIPs – provided the research team with the information on the most frequently spoken languages in AIPs in the period when data collection was planned. The translations were made by native speakers (Arabic, Farsi and Pashtu).

####  Sampling Scheme

 Initially, participants would have been selected through a probability sampling scheme to ensure that representative, generalizable and significant estimates could be made regarding SV victimisation and perpetration in the AIPs population residing in Belgium.^12,14-16^ To obtain reliable results, we planned to interview approximately 400 AIPs: 200 in Flanders and 200 in Brussels and Wallonia. The initial power calculation was based on an estimated victimisation rate of 57%, as was found in the study by Keygnaert et al^17^ and the number of AIPs residing in Belgium at that time (ie, August-September 2016). To have some extra margin, we rounded up the targeted sample size to 400 (see Table). Taking into account non-response and refusal to participate due to the sensitivity of the topic, we estimated that up to 4 times as many people would have to be contacted to ultimately reach the targeted sample size.

**Table:** Sample Size Calculation

	**Original Power Calculation**
Z-value for *P* <.05	1.96
Proportion (P)	0.57
Margin of error (E)	0.05
N total	377

 The Waiting Register of the Belgian National Register would serve as the sampling frame. The Belgian National Register serves as a database containing specific legal information which makes it possible to identify natural persons living in Belgium.^18^ The Waiting Register is kept in every municipality and contains the data of those foreign persons who declare themselves as refugees or who ask to be recognized as refugees, or as AIPs. This register is centralized by the Belgian National Register, making it possible to access information on all AIPs.^18^ Hence, individuals unrecorded in this registry, such as undocumented migrants or homeless people, could not be integrated in the sampling frame. In the initial research design – approved by the donor - the research team would have been able to invite randomly selected potential participants - about 200 AIPs in Flanders and 200 AIPs in Brussels and Wallonia – via the Waiting Register. After the random sample would have been drawn by the Waiting Register, the register would have provided the researcher with contact information for the selected potential participants. As such, selected AIPs would have been contacted directly to ask for their willingness to participate and to arrange an interview.

###  The Research Strategy Beyond GDPR

**Figure 1:**
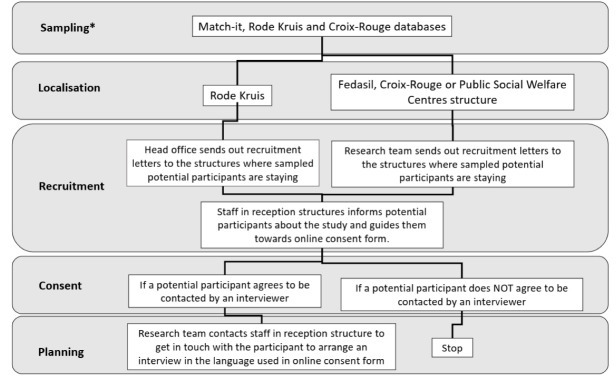
Schematic Overview of the Recruitment Process. Note: Inclusion criteria: relevant legal status (ie, AIP), age (ie, +16 years old), and nationality (ie, 142 nationalities from countries were at least English, French, Dutch, Arabic, Dari, Farsi or Pashtu is spoken).

 Due to the implementation of the GDPR legislation, inviting AIPs directly via the Waiting Register was no longer possible. In concertation with national and international experts involved in the scientific guidance committee of the UN-MENAMAIS project, it was decided to apply a new recruitment strategy (see Figure 1). This new recruitment strategy was presented for ethical approval to the Commission for Medical Ethics of Ghent University Hospital and was approved on the 21st of May 2019.

####  Sampling

 In this new strategy, the Match-It database^19^ from Fedasil served as the main sampling frame. In Belgium, the reception facilities for AIPs comprise collective and individual reception structures.^20^ The individual reception structures or ‘local reception initiatives,’ are housing organised by the Public Social Welfare Centres and non-governmental organization’s. The collective structures or ‘reception centres’ are managed by Fedasil, the Red Cross of Belgium or other partners.^20^ The Red Cross of Belgium is regionally organised as “Rode Kruis – Vlaanderen (Rode Kruis)” for the Flemish region and “Croix-Rouge de Belgique, Communauté francophone (Croix-Rouge)” for Wallonia and Brussels. The Match-it database is the online database that contains information about AIPs in Belgium in function of the management of both collective and individual reception places and residents (usually AIPs) in the Belgian reception network.^19^

 During the period of implementation of the new recruitment strategy (July 2019), the Match-It database was still under development. AIPs staying in reception initiatives of the Rode Kruis and Croix-Rouge were expected to only be included in this database as of the second half of 2019. As a result, a proportionate number of AIPs had to be randomly selected from the internal Rode Kruis and Croix-Rouge databases as well using the same sampling criteria: 65% of the AIPs were selected from the Match-It database and 35% from the Rode Kruis and Croix-Rouge databases. These percentages reflect the spread of assigned AIPs to centres from these three partners. As the integration of these databases into the Match-it database had not been completed by the end of 2019, the following waves in 2020 had to be drawn in the same manner from these 3 main registers.

 Because of the GDPR legislation, selected potential respondents for interviews could no longer be contacted without their permission and as such could no longer be invited for participation directly. Consequently, drawing the lists with selected AIPs from the existing databases had to be done in an encrypted manner. In the initial stages of recruitment, the research team did not have access to the personal data of the potential respondents in order to provide maximum protection of privacy.

 The head offices of the reception structures (Match-It, Rode Kruis and Croix-Rouge) have drawn a random sample, based on the criteria provided by the research team, such as legal status, age, gender and nationality. Since ‘language’ was not included in the databases, nationality was used as a proxy to sample respondents who were likely to speak one of seven languages. The 142 nationalities included were based on list of countries were at least one of the languages (English, French, Dutch, Arabic, Dari, Farsi or Pashtu) is spoken.

####  Localisation

 The researchers received three lists of unique codes that corresponded to respective potential participants: one from Fedasil based on the Match-it database, one from Rode Kruis and one from Croix-Rouge. The unique code could be used to identify where the potential respondent was staying. This could be in either a reception centre or a local reception initiative.

####  Recruitment

 Once the reception structures hosting the potential participants were identified, they were contacted by the research team or their head offices in the case of Rode Kruis. The reception structures were asked to act as an intermediary in providing the information about the study and an online informed consent form for participation to the selected potential participants. The staff from the reception initiatives could use the unique code to identify the selected potential participant and make contact with that person to inform them about the study.

####  Consent

 To make the information transfer as accurate as possible and to avoid selection bias, a standardized online information letter and online consent form were used. In this way distorted or incomplete information transfer through a third party was avoided. In addition, the intent was to minimise social desirability effects and to guarantee the safety of the respondent. Previous studies on this topic in the European asylum sector taught the researchers that participation in such studies may bring participants into trouble with the communities they belong to. When some communities learned about the nature of these studies, they forbade the participant from continuing their participation or even pressured/ threatened them into changing their testimonials.^21^ By using a standardized online information letter and online consent form we tried to avoid these types of situations.

 However, in the target population of the study there is a high degree of lower levels of literacy.^5-8^ Working with a standardized online information letter alone could therefore lead to the identification of only a subpopulation of the target population. For this reason, an audio version of this information letter and a permission module were introduced to the website (cf. infra). The potential participant could thus confirm or refuse their participation in a non-written way as well.

**Figure 2:**
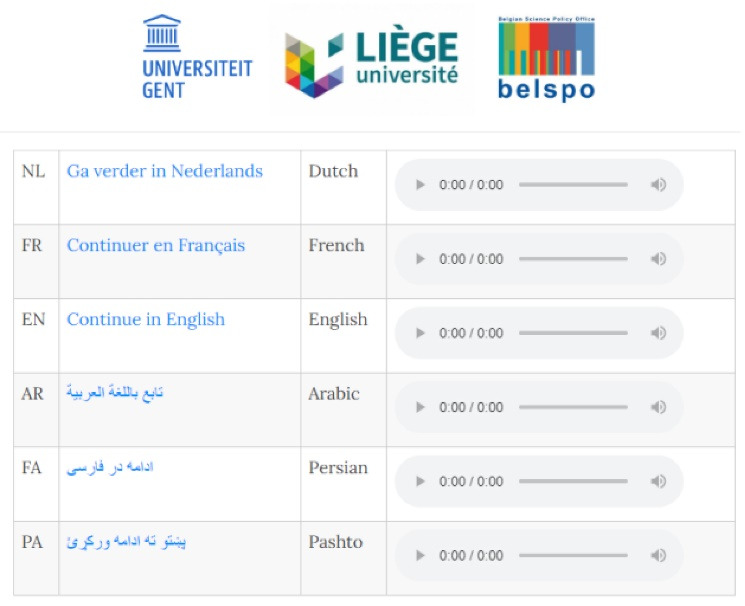
Welcome Page on the Website http://gsw2019.netlify.app.

 We developed a multilingual website that could be consulted via smartphone or computer available to AIPs at the place of residence on which the information letter and the consent forms were integrated. Both were recorded by native speakers in the relevant languages and were accessible on the website both in a written and audio version. Less literate AIPs could listen to the information and follow the instructions to indicate their preference (see Figure 2).

 A letter with a link to a webpage was sent to the social service of the place of residence of the selected potential participant. This letter contained instructions for the social service and an individualized letter for the potential participant with an individualized code and link to the website. This code was unique to every potential participant (UG001, UL001, etc).

 The reception structure’s staff was instructed to guide the potential participant to the website and if needed to help them with selecting the desired language and form (written or audio) of the information letter and consent module. Before the potential participant could start the process, they had to be left alone by the staff member to avoid social desirability bias.

 To avoid participants mis-transcription, we designed the personal link so that each personal code was automatically filled in the right field when following the URL.

 After listening to/reading the information letter, the potential participant could choose whether or not to agree to be contacted for participation.

 Because the potential participant entered a personalized code, the research team received automatically a confirmation about which participant (by means of the corresponding individualized code) may or may not be contacted. Based on this, the contact details of the respondent could then be requested from Fedasil, Rode Kruis, Croix-Rouge or the Public Social Welfare Centre.

 It should be highlighted that at this stage the AIP did not yet agree to participate in the interview. Consent to participate in the study was only officially given at the moment the interviewer discussed the information letter and informed consent file prior to starting the interview. If the potential participant refused participation at that stage, no data would be collected.

####  Planning

 Only after the reception of the potential participant’s online consent, their personal data was shared by the reception structure with the research team to plan the interview. In collaboration with the staff working in reception centres and local reception initiatives, interviews were planned with possible respondents and an interviewer speaking the corresponding language. The interviews would take place in a separate room where the interviewer and interviewee would ideally not be disturbed during the course of the interview.

## Results

 Between the second half of September 2019 when the first invitations were sent out to the place of residence of the randomly selected AIPs and the beginning of March 2020, in total 1626 AIPs had been sampled. Of those, 811 AIPs had been informed about the study via the reception structures, 330 AIPs had given an answer on the website about their willingness to participate in an interview, 217 AIPs agreed to be contacted by the research team and 62 AIPs had been interviewed. This means that although the subject of the study is sensitive, AIPs seem to be willing to participate in this type of research. Unfortunately, our study had to be stopped before we reached the target of 400 AIPs due to the Covid-19 containment measures taken by the Belgian government in the beginning of March 2020.

## Discussion

 Conducting research on SV in AIPs goes hand in hand with a number of research challenges, particularly when the aim is to conduct a high-quality prevalence study (see De Schrijver et al^1^ for a more detailed discussion). Below, we will briefly describe the challenges we were faced with while implementing the new procedure and the lessons we have learned. Based on our experiences, we give some recommendations for future studies in hard-to-reach populations such as AIPs.

 Overall, the new recruitment procedure required an intensification of the project coordination. It required more steps in the process of getting access to the randomly selected potential participants, planning and conducting the interviews. Every step that was added to the procedure in comparison to the original strategy held the risk of introducing various biases, including a selection and non-response bias. As such, every possible option or alternative was weighted but could not be followed as they were deemed too prone to introduce additional biases.

 To begin with, to be able to access the 3 databases from Fedasil (Match-it), Rode Kruis and Croix-Rouge, strong partnerships were needed. The new way of sampling the potential respondents required these partners to be closely involved in the project which created an additional workload for their staff. If one of these instances would not have been convinced of the pressing need to collect data on SV in AIPs in Belgium and be willing to share their database with the research team, a randomized sample reflecting AIPs staying in different kinds of reception initiatives in various regions of the country could not have been drawn. In addition, the head offices of Fedasil, Rode Kruis and Croix-Rouge played an important role in the development of the recruitment strategy. First of all, they gave feedback on the proposed procedure, indicated what the strengths and weaknesses could be and were active in finding alternatives. Further, they also played an important role in motivating the different reception initiatives to facilitate the study. For example, the response rate of AIPs residing in centres from one of the reception structures was higher than in the centres from other initiatives. This may have been the result of the structured way in which the first reception structure asked their staff to follow-up on the recruitment. In contrast to the other types of centres, the staff received the recruitment letters directly from the head office as well as the instruction to approach the randomly selected AIP within a timeframe of two weeks. In future studies, applying a comparable structured approach could help to increase the response rate and time efficiency. Moreover, using a common strategy in all types of reception centres is essential to avoid selection bias.

 In addition, a strong involvement from the staff working in reception centres and local reception initiatives is also essential. Given the high pressure staff members experienced at this moment in the asylum sector, helping researchers cannot always be set as a priority. This was further emphasized by the reception structures as occupancy rates in the centres reached a peak around the end of 2019 again.

 It is important to highlight here that the dependence of the research team on the preparedness of the reception initiatives to collaborate in the data collection is a vulnerability. These initiatives function as gate keepers and may block access to the research population.^1^ Investing in good working relationships is therefore crucial to make this type of studies possible. However, researchers should also be aware that investing in these close working relationships may influence their objectivity and may create bias when evaluation of the context in which AIPs are staying, of the working of the initiatives and of the policies guiding them is part of research objective.

 Coordination was also intensified within the research team itself. First, the development of the website, the translation of the additional text for the website into different languages and the recording of the information texts in those languages was not foreseen in the initial project plan. This resulted in more working hours than foreseen on this work package and in higher costs for translation.

 Secondly, the new procedure required a close follow-up of the database with answers via the website from potential participants, the schedules of the interviewers, the contacts with the staff of the reception structures and the planning of interviews. In our experience the more time passed between the moment potential participants were informed about the study and the moment the interview could be planned, the higher the chance that potential participants changed their minds or had left the reception structure and could not be contacted anymore. It is thus crucial to limit that time as much as possible to avoid participation reduction.

 Due to GDPR, access to data about an AIP’s nationality, education level and mother tongue was not possible. The research team only knew the AIP was 16 years or older, his or her internal identification code within the Match-it database or the databases from Rode Kruis and Croix-Rouge, the place of residence and that the AIP had claimed in his or her application for international protection to come from a country where Dutch, English, French, Dari, Farsi, Pashtu or Arabic was spoken. This information was based on the inclusion criteria used in the sampling frame. Researcher realised that using nationality as a proxy to spoken language entailed the possible risk of introducing bias. In some countries, certain national languages are only spoken by higher educated citizens and local languages may not be recognised as national languages. However, the objective of this study was to estimate the prevalence of SV in the total group of AIPs residing in Belgium. Moreover, the survey contained questions regarding socio-economic status, educational level etc. to be able to identify those factors that increased the risk for SV even more in AIPs. The researchers considered using nationality as a proxy to language for this study to be the best option to grasp the diverse group of AIPs in Belgium, but recognise that future studies should consider potential other proxies as well.

 After accessing the website with the consent module (cf. supra), the researchers could guess the language an AIP spoke based on the language they used to provide the answer to participate in an interview. In a number of cases, after an interviewer made an appointment with the respondent via the reception initiative, the information the research team had received from the reception initiative about the respondent, appeared to be incorrect. This sometimes meant that a new appointment had to be made with another interviewer to match the languages. Moreover, interviewers sometimes had to make more than one appointment per respondent because the information about their availability had changed. In general, this study required more work-hours from the research team than foreseen in the original project plan.

 When designing this study, the average time of residence was difficult to estimate as there were more persons applying for international protection and entering the system than AIPs were leaving the system. The longer processing time of asylum cases prolonged the stay in reception centres. As a result, and also due to the drastic reduction of reception places in 2017-2018, the reception sector was placed even more under pressure. The time between initialisation of the application procedure and the actual interview(s) related to their application, varied depending on the situation of the AIP, the reason for applying for international protection, the country an AIP came from etc. The researchers did not receive specific information regarding the average time of residence in the centres but based on informal information exchanged by the sector, the research team knew that this period could be months up to years for some. Further, the researchers did not receive any information on the application stage potential participants were in. Given that we had foreseen to leave an absolute maximum of 10 months between the moment of sampling and the structured interview and even tried to limit this period to less than 3 months, we initially did not expect major problems with drop-out related to this. However, the complexity of the coordination of the recruitment procedure led to a significant delay in time between the first invitation of the respondents to access the consent website and the moment they were invited for the actual interview, which subsequently led to a major loss of follow-up. Many AIPs who initially indicated to be willing to participate in an interview had either left the place where they were staying and could not be contacted anymore or had lost interest to participate. Because of GDPR, we could not trace them without violating their right to privacy.

 In some reception centres, only a few or almost no selected AIPs gave an answer on the website. When this pattern became clear, the researchers contacted the centres to verify whether the methodology and the protocol were followed. The loss of follow-up may thus also partly be related to a misunderstanding of the procedure by the staff in reception initiatives. While contacting the centres, the research team learned that the availability of internet connection and computers was unequally present in the different reception centres. In addition, not all respondents had a smartphone or computer skills, therefore the help of social workers was needed.

 During the recruitment of the first wave, the research team learned much about the practical implementation of the new strategy. Future studies should foresee sufficient time and resources to not only pilot the questionnaire to be used, but also the recruitment strategy.

 In the end, a much bigger sample would have been necessary to reach the target of 400 interviewed AIPs. Initially we calculated that 4 waves of 200 respondents in Flanders and 200 in Wallonia would be sufficient to reach the objective. However, with the application of the new sampling and recruitment strategy we expected to need at least 3 waves of 400 participants in Flanders and 3 waves of 400 participants in Wallonia. Unfortunately, it is currently impossible to give a more sensitive estimation of how big the recruitment sample should be in future studies given that we had to stop the data collection before we reached our target of 400 participants due to the coronavirus disease 2019 (COVID-19) pandemic in March 2020.

 Based on our experiences with this study, we would like to formulate some recommendations for future studies aiming to include a representative sample of AIPs. Firstly, as is ethically sound, design the study together with the head offices of the reception structures. Secondly, as a result of the GDPR, relying on national registers for drawing randomized samples may no longer be possible for certain populations. As such, it may be fruitful to explore other databases (eg, from national reception initiatives) as starting points. In addition, it may also be useful to explore whether random sampling is the best approach for reaching a representative sample of the hard-to-reach population you want to study. Depending on the research objective, cluster sampling, respondent driven sampling, or time-location sampling may in some cases prove to be a more fitted strategy. In the case of study – which is part of a larger national representative study on SV, we opted for simple random sampling to be able to collect robust prevalence data in AIPs that could be compared to the data collected in the general population. In the case of our study, we judged that simple random sampling would result in the best comparable data. In previous studies,^21^ researchers had the experience that potential participants to studies on sexual health and violence were dissuaded by other participants when the purpose of the study became clear. This type of selection bias linked to working with seeds – as is central to respondent driven sampling – would have compromised the representativeness of our data significantly. Further, time-location sampling was also less suited for this particular study as this would have required even more time, budget and involvement from the reception centres staff to organise this type of sampling and map the “venue-day-time” increments which form the unit of random sampling in time-location sampling.^22^ However, the use of other sampling strategies may also allow to include other vulnerable and hard-to-reach groups such as homeless people and undocumented migrants.

 In addition to the standardized online information letter and online consent form as it was designed for this study, it could be useful to ask potential participants immediately their preferred language for the interview to avoid a potential mismatch in language between the interviewer and interviewee.

 Next, we recommend to foresee sufficient budget for translations and audio recordings. Since the AIP population is a constantly changing one by nature, the composition may modify very quickly and unexpectedly and as a consequence so may the most frequently spoken languages within this group. As a result, translations will need to be made as close to the start of the recruitment of participants as possible. In order to get high quality work with high flexibility in terms of timing and requested languages, it is recommended to work with professional translation partners, which comes at a higher cost than working with volunteers who are fluent in the frequently spoken languages. In addition, it is recommended to ask for a (back-)translation of materials regarding sensitive topics such as sexuality to verify whether translations are made literally or by means of euphemisms. As such, one can avoid interpretation bias introduced via translations.

 Last but not least, we recommend hiring staff specifically assigned to the recruitment process of the study. In our study, due to heavy budget costs, we had to rely heavily on the staff in the reception initiatives. Given the high pressure the asylum reception sector is under, it is recommended to take the different steps in the recruitment process in your own hands as much as possible in order not to burden the staff in the centres. Therefore, good working relations with the staff in centres is essential. Moreover, by letting research staff approach the potential respondents after they have been sampled in advance, one avoids gatekeeping effects and an inaccurate transfer of information about the study. Recruiting potential participants can be made much more standardized. In addition, the research staff may bring laptops or tablets with an offline information letter and consent file in the different languages to avoid lower response rates in centres where access to the internet is limited. The use of offline forms may in turn also add to the standardisation of the recruitment procedure.

 Overall, conducting prevalence studies on SV in AIPs beyond GDPR requires more intensive coordination, revised budgets and larger research teams.

## Conclusion

 The case of the UN-MENAMAIS study illustrates how designing a research strategy including a nationwide randomized sample of AIPs while respecting ruling legal frameworks, ethical considerations and high scientific standards may ultimately result in an approach that is more complex than before. Given the challenges we encountered while implementing the new sampling frame and recruitment strategy, we believe that conducting prevalence studies on SV in hard-to-reach populations such as AIPs remains possible but requires a very intensive project coordination and considerate budget. In conclusion, to achieve high-quality prevalence numbers on SV in AIPs while respecting the new GDPR regulations, studies will require an approach that has become significantly more time consuming and resource-intensive to implement.

## Ethical issues

 This study was performed in line with the principles of the Declaration of Helsinki. The UN-MENAMAIS project was approved by the Commission for Medical Ethics of Ghent University Hospital on the 21st of November 2018. The amendment concerning work package 2 on AIPs was approved by the same commission on the 21st of May 2019.

## Competing interests

 Authors declare that they have no competing interests.

## Authors’ contributions

 LDS performed the project administration, conceptualization, methodology, investigation, training of interviewers, software and writing–original draft. ACI and BH contributed in methodology, investigation, training of interviewers, writing–review and editing. CV contributed in conceptualisation, acquired the research funding, writing–review and editing. LN contributed to supervision, conceptualization, acquired the research funding, methodology, writing–review and editing, IK coordinated the project, acquired the research funding, trained the interviewers and performed supervision, conceptualization, methodology, writing–review and editing.

## Funding

 This work was supported in part by the Belgian Science Policy Office - Belgian Research Action through Interdisciplinary Networks funding scheme [BR/175/A5/UN-MENAMAIS to CV, LN, IK]. CV’s contribution to this work was supported in part by the Research Foundation—Flanders (FWO) Postdoctoral Fellowship funding scheme [12C0619N to CV].
